# Neurovascular Coupling of the Posterior Cerebral Artery in Spinal Cord Injury: A Pilot Study

**DOI:** 10.3390/brainsci3020781

**Published:** 2013-05-08

**Authors:** Aaron A. Phillips, Andrei V. Krassioukov, Mei Mu Zi Zheng, Darren E.R. Warburton

**Affiliations:** 1 Cardiovascular Physiology and Rehabilitation Laboratory, Physical Activity Promotion and Chronic Disease Prevention Unit, University of British Columbia, Vancouver V6T 1Z1, Canada; E-Mails: aaron.phillips.ubc@gmail.com (A.A.P.); meimuzizheng@hotmail.com (M.M.Z.Z.); 2 Experimental Medicine Program, Faculty of Medicine, University of British Columbia, Vancouver V6T 1Z1, Canada; 3 International Collaboration of Repair Discoveries, University of British Columbia, Vancouver V6T 1Z1, Canada; E-Mail: andrei.krassioukov@vch.ca; 4 Division of Physical Medicine and Rehabilitation, Department of Medicine, University of British Columbia, Vancouver V6T 1Z1, Canada

**Keywords:** spinal cord injury, neurovascular coupling, posterior cerebral artery

## Abstract

Purpose: To compare neurovascular coupling in the posterior cerebral artery (PCA) between those with spinal cord injury (SCI) and able bodied (AB) individuals. Methods: A total of seven SCI and seven AB were matched for age and sex. Measures included PCA velocity (PCAv), beat-by-beat blood pressure and end-tidal carbon dioxide. Posterior cerebral cortex activation was achieved by 10 cycles of (1) 30 s eyes closed (pre-stimulation), (2) 30 s reading (stimulation). Results: Blood pressure was significantly reduced in those with SCI (SBP: 100 ± 13 mmHg; DBP: 58 ± 13 mmHg) *vs.* AB (SBP: 121 ± 12 mmHg; DBP: 74 ± 9 mmHg) during both pre-stimulation and stimulation, but the relative increase was similar during the stimulation period. Changes in PCAv during stimulation were mitigated in the SCI group (6% ± 6%) *vs.* AB (29% ± 12%, *P* < 0.001). Heart rate and end-tidal carbon dioxide responded similarly between groups. Conclusions: Clearly, NVC is impaired in those with SCI. This study may provide a link between poor perfusion of the posterior cerebral region (containing the medullary autonomic centres) and autonomic dysfunction after SCI.

## 1. Introduction

Spinal cord injury (SCI) leads to not only devastating paralysis, but also serious cardiovascular complications [1,2,3,4]. Recently, cardiovascular disease has been identified as a primary cause of death in those with SCI [5]. Cerebral vascular disease is of major concern, as those with SCI have a two to three-fold increase in the risk of stroke (particularly ischemic stroke) [6]. 

Cerebrovascular reserve is an important marker of the relationship between blood pressure and cognitive function. A reduction in cerebrovascular reserve has been associated with impaired neurovascular metabolic coupling (NVC) and reduced cognitive function [7]. Neurovascular coupling refers the coupling of brain metabolism and blood flow in the human cerebral circulation [8] involving the interactions between blood vessels, neurons, and other nervous system cells (e.g., astrocytes and other glial cells) [7]. Neurovascular coupling works in a synchronized manner to match (on a beat-by-beat basis) neuronal activity and perfusion [7]. The continuous assessment of cerebral blood flow velocity (CBFv, a surrogate of cerebral blood flow) via transcranial Doppler ultrasonography [9] allows for the non-invasive evaluation of NVC during cognitive tasks [8,9]. Measuring NVC has been shown to be useful for identifying subtle brain impairment in a variety of clinical populations including schizophrenia, Alzheimer’s disease, hypertension, stroke, and epilepsy [10,11,12,13,14,15,16,17,18].

The medullary region of the brainstem is the primary centre of autonomic control. Poor cerebrovascular regulation after SCI in the medullary region may exacerbate blood pressure and autonomic instability commonly found in this population [19,20]. Blood flow delivery to the medullary region of the brain is a combination of arterial branches (*i.e.*, anterior spinal artery, posterior inferior cerebellar artery) deriving from the vertebral arteries. As the posterior cerebral artery (PCA) arises (in the majority of people) from the vertebral arteries, an evaluation of PCA function in those with SCI is pertinent to our understanding of autonomic dysfunction (as it relates perfusion of the autonomic brain centre) in this population. 

Our study aimed to measure PCA regulation in those with high- level SCI (*i.e.*, >T2) by quantifying NVC in this population as compared to age- and sex-matched able-bodied controls (AB). Due to a combination of low resting blood pressure and impaired sympathetic control of cerebral blood vessels (due to SCI within or above levels of superior cervical ganglia), we hypothesized that individuals living with SCI would have impaired NVC as compared to able-bodied controls. 

## 2. Methods

Seven individuals (5 males) with SCI (C_4_-T_1_, ASIA A, B) participated in this study (Table 1). Time since injury ranged from (7 to 324 weeks) and all participants were inpatients at GF Strong Rehabilitation Hospital in Vancouver, Canada (*n* = 7). The control group (AB) was comprised of 7 participants (5 males) matched for age (SCI: 33 ± 12 *vs.* AB: 31 ± 11 years) and sex. Participants were similar for height (SCI: 173 ± 7 *vs.* AB: 173 ± 11 cm), weight (SCI: 66 ± 12 *vs.* AB: 69 ± 15 kg) and body mass index (SCI: 22 ± 3 *vs.* AB: 23 ± 3 kg/m^2^). 

**Table 1:** Cerebral blood velocities and cardiorespiratory measures pre- and post-visual stimulation and peak percent PCAv response. * *P* < 0.05 between AB (able bodied) (*n* = 7) and SCI (spinal cord injury) (*n* = 7).

	Pre-stimulation	Value at peak-response	Peak percent change CBFv (%)
**PCAv (cm/s)**			
AB			
Peak Systolic	59 ± 10	72 ± 13	24 ± 7
End Diastolic	22 ± 10	34 ± 5	12 ± 6
Mean	37 ± 6	47 ± 7	29 ± 12
SCI			
Peak Systolic	57 ± 16	59 ± 14	6 ± 7 *
End Diastolic	27 ± 9	29 ± 8	10 ± 4 *
Mean	37 ± 10	38 ± 7	6 ± 6 *
**Cardiorespiratory Metrics**			
AB			
PETCO_2_ (mmHg)	33.6 ± 3.9	32.6 ± 3.7	−2.9 ± 2.3
MAP (mmHg)	90 ± 6	92 ± 6	3 ± 2
HR (beats/min)	73 ± 10	75 ± 11	4 ± 3
SCI			
PETCO_2_ (mmHg)	33.1 ± 4.1	31.3 ± 4.9	−4.9 ± 5.0
MAP	72 ± 12 *	74 ± 11 *	3 ± 3
HR	75 ± 23	77 ± 24	4 ±4

All participants were instructed to abstain from caffeine, exercise, and alcohol for 24 h prior to testing. Those who were smokers or had any history of CVD were excluded from participation. Participants were tested in the morning after an overnight fast and abstained from all medications for that day until after completion of testing. For the SCI participants, testing took place in their hospital room in the morning. All participants provided written informed consent conformed to the standards set by the *Declaration of Helsinki* and as approved by the Clinical Research Ethics Board at the University of British Columbia. 

### 2.1. Experimental Protocol

Our team arrived and transferred the participants to their wheelchair if they were capable (*n* = 5), or moved the mechanized hospital bed into the seated position (*n* = 2). For AB participants, testing took place in the morning within a dedicated research space in the same location. Both AB and SCI rested in the seated position for 30 min prior to starting the NVC procedure. 

Electrocardiography, expired end-tidal carbon dioxide (ETCO_2_ (AEI Technologies, Pittsburgh, PA)) from a leak-free mask, and non-invasive beat-to-beat blood pressure (via photoplethysmography (Finometer, TPD Biomedical Instrumentation)) were collected at 1 kHz per channel through an analog-to-digital converter (16 channel Powerlab, ADInstruments, Colorado Springs, CO, USA). 

The P1 segment of the right PCA was isonated via a 2 MHz transducer placed on the temporal window (Spencer Technologies, Seattle, USA). The PCA blood velocity (PCAv) was collected and recorded through the analog-to-digital converter. The PCA was confirmed using ipsilateral carotid compression that causes an increased velocity in the PCA and a velocity reduction in both the middle and anterior cerebral arteries [9].

The NVC procedure included 10-repeated bouts intended to activate the visual cortex. Each bout consisted of a 30 s pre-stimulation period where the participant sat quietly with eyes-closed, followed up a 30 s long stimulation period where the participants read from a general interest magazine. The same magazine was used for all participants. The participants were provided an auditory stimulus every 30 s with either the command “eyes open” or “eyes closed” while a research assistant held the magazine at an appropriate height and distance. One practice trial occurred prior to the 10 cycles. Using the electrocardiogram to gate the extraction, beat-to-beat heart rate, peak systolic and end diastolic blood pressure, as well as peak and end diastolic PCAv were recorded continuously. Also, the peak expired carbon dioxide value was exported from each breath to measure ETCO_2_.

### 2.2. Data Analysis

All values were interpolated using cubic spline at 0.2 Hz. All 10 trials were aligned according to the initiation time (eyes open) and averaged to generate one response slope per subject. Maximum and minimum (ETCO_2_) values were extracted (using a customized Microsoft Excel program) and evaluated statistically. All comparisons were evaluated using an independent t-test between groups with alpha set to 0.05. Data are presented as means ± SD. 

## 3. Results

Resting seated blood pressure was significantly lower in the SCI group *versus* AB (SBP: 100 ± 13 *vs.* 121 ± 12 mmHg, respectively; DBP: 58 ± 13 *vs.* 74 ± 9 mmHg, respectively). The difference in blood pressure between the two groups remained throughout the stimulation period; however, the change occurring from pre-stimulation to stimulation was similar (ΔSBP: SCI 3% ± 3% *vs.* AB 3% ± 2%; Table 1). Heart rate was similar between groups (Table 1). The ETCO_2_ did not change significantly from pre-stimulation to stimulation in either group. 

**Figure 1:**
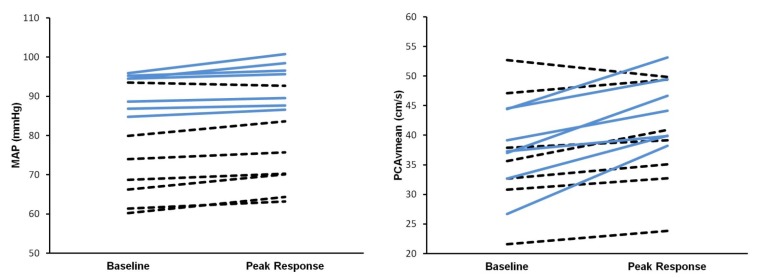
Individual data for mean arterial pressure (MAP; left) and posterior cerebral artery mean blood flow velocity (PCAv_mean_; right) pre- and post-visual stimulation. Black hash-lines represent those with SCI (spinal cord injury). Blue solid slides represent able-bodied individuals.

The average pre-stimulation PCAv was similar between the AB (37 ± 6 cm/s) and SCI (37 ± 10 cm/s) participants (Table 1). The increase in mean PCAv during the stimulation period was significantly lower in SCI (6% ± 6%) as compared to AB (29% ± 12%). Similarly, increases in both systolic and end-diastolic PCAv during the stimulation task were reduced in the SCI group (Table 1). Individual data is presented in Figure 1.

## 4. Discussion

This is the first study to report NVC in SCI. We have shown that NVC is impaired in those with SCI. Specifically, the PCAv response to posterior cortex stimulation is mitigated in those with SCI. As the PCA is directly downstream from the vertebral arteries (which provide the majority of blood flow to the medullary autonomic centres in the brain) this finding may have important implications for autonomic dysfunction after SCI [21]. These findings also highlight the need to engage in interventions (such as exercise training) that are known to improve both cerebrovascular and cognitive function. 

We are not aware of any studies examining PCA blood flow in those with SCI. Similar resting PCAv in both groups suggests the static cerebral autoregulatory capacity of the PCA is intact in those with SCI. The 29% ± 12% increase in the PCA in AB is comparable to that reported elsewhere using a similar stimulation task [8]. The lack of an apparent NVC response in those with SCI may be due to an array of systemic and/or cerebral factors. From a systemic perspective, MAP is reduced in those with SCI, especially those with high level injuries as studied presently [22]. Cerebrovascular reserve described the ability of the brain blood vessels to provide increases blood flow in response to increases metabolic or chemical stimuli [15]. As such, maintenance of PCAv in our SCI group, who are markedly hypotensive, represents an increase in cerebrovascular conductance and potentially a decrease in cerebrovascular reserve. We feel it is likely that intact static cerebral autoregulation (*i.e.*, increased cerebrovascular conductance in order to maintain PCAv during hypoperfusion) in our SCI group impaired cerebrovascular reserve and severely disrupted the ability of the brain to further increases conductance in response to increased metabolic stimuli. In other words, it may be that the perfusion pressure in those with SCI is too low to make use of small and subtle changes in vasomotor tone that occur during increased cerebral metabolic demand [23,24]. Indeed, Duschek and Schandry showed that NVC of the middle cerebral artery was reduced in non-SCI individuals with hypotension similar to those shown in our SCI group [25]. In contrast, work by Willie et al. showed that NVC of the PCA was similar at rest and during exercise (when blood pressure was elevated); suggesting underling arterial pressure does not influence the NVC response [8]. Persons living with SCI have decentralization of descending sympathetic fibres [22]. This leads to not only resting hypotension, but also aberrant blood pressure responses [19]. The inability of those with SCI, at injury levels studied in this experiment, to acutely alter blood pressure may help explain the severely reduced NVC response in SCI. This certainly does not tell the whole story however, as although resting blood pressure was lower in SCI, the relative blood pressure response to stimulation was similar to AB; suggesting acute increases in perfusion pressure during cognition may not be the primary factor mediating PCAv responsiveness. 

Alternatively, sympathetic vasomotor control in the brain is achieved through the superior cervical ganglion which receives input from the T_1_ to T_4_ spinal nerves [26]. It is likely that central cerebrovascular sympathetic control was severely impaired in our SCI group whom had sustained injuries from above the T_1_ level in all but one case. Prior work in those with autonomic failure supports this contention, showing a similar blood pressure response to cognition with a mitigated cerebral blood flow response [27]. Accordingly, if sympathetic control plays a role in NVC, the loss of sympathetic control over cerebrovascular tone is likely to be partially responsible for the reduced NVC response shown in our SCI group. 

An important limitation of this study arises from the use of trancranial Doppler for estimation of blood flow. Because of the attenuation of the ultrasound signal when travelling through the skull, transcranial Doppler can only estimate flow velocity. As per convention and supported by evidence in humans, using hypovolemic stimulus causes no change in middle cerebral artery diameter; we infer a constant diameter of the PCA [28,29]. 

## 5. Conclusions

The results of our study demonstrate those with SCI have reduced NVC as compared to AB. This may be due to impaired sympathetic cerebrovascular control as well as low resting systemic blood pressure. As the PCA is directly related to effective perfusion of the medullary autonomic centres in the brain, our study may highlight an important connection between impaired cerebral perfusion of the brainstem and autonomic dysfunction after SCI (*i.e.*, impaired blood flow delivery influencing impaired medullary function). These findings have important implications for addressing the altered cerebrovascular control seen in SCI. Interventions (such as exercise training) that are known to enhance both cerebrovascular and cognitive function may be particularly important for persons living with SCI to reduce the risk for secondary complications (such as cerebrovascular disease) and the development of cognitive impairment. 
